# Upregulation of neuronal PGC-1α ameliorates cognitive impairment induced by chronic cerebral hypoperfusion

**DOI:** 10.7150/thno.37119

**Published:** 2020-02-03

**Authors:** Bin Han, Wei Jiang, Haijie Liu, Junjie Wang, Kai Zheng, Pan Cui, Yan Feng, Chun Dang, Yali Bu, Qing Mei Wang, Zhenyu Ju, Junwei Hao

**Affiliations:** 1 Department of Neurology, Xuanwu Hospital, Capital Medical University, Beijing, 100053, China; 2 Department of Neurology, Tianjin Medical University General Hospital, Tianjin, 300052, China; 3 Department of Neurology, The Affiliated Hospital of Qingdao University, Qingdao, 266000, China; 4 Stroke Biological Recovery Laboratory, Department of Physical Medicine and Rehabilitation, Spaulding Rehabilitation Hospital, the teaching affiliate of Harvard Medical School, Charlestown, MA, 02129, USA; 5 Key Laboratory of Regenerative Medicine of Ministry of Education, Institute of Aging and Regenerative Medicine, Jinan University, Guangzhou, 510632, China

**Keywords:** neuroinflammation, PGC-1α, ROS, vascular dementia

## Abstract

**Rationale**: Mitochondrial dysfunction and oxidative stress occur in vascular dementia (VaD), but the specific molecular mechanism regulating these events remains unclear. Peroxisome proliferator-activated receptor-γ co-activator-1α (PGC-1α) is a master regulator for mitochondrial function. This study aims to investigate whether PGC-1α is involved in the pathophysiology of VaD.

**Methods**: We firstly generated *PGC-1α*^f/f^ Eno2-Cre mice to induce neuron-specific overexpression of PGC-1α by crossbreeding *PGC-1α*^f/f^ mice with Eno2-cre mice. Then, the mice were subjected to bilateral common carotid artery stenosis to induce chronic cerebral hypoperfusion. Neurological function and hippocampal PGC-1α expression was evaluated. Next, RNA-Seq analysis and Seahorse assay were performed on the hippocampal neurons. In addition, mitochondrial antioxidants, uncoupling proteins, ROS production and the activation of glial cells were also measured.

**Results**: Our results showed that hippocampal PGC-1α expression is down-regulated in the mouse VaD model induced by chronic cerebral hypoperfusion. In contrast, neuronal PGC-1α overexpression significantly ameliorated cognitive deficits. RNA-Seq analysis indicated that PGC-1α improved energy metabolism of neurons under hypoxic condition, and Seahorse assay confirmed that PGC-1α increases the metabolic activity of neurons. Further study demonstrated that PGC-1α boosted the expressions of mitochondrial antioxidants and uncoupling proteins (UCPs), including SOD2, Prx3, GPx1, UCP2, UCP4 and UCP5, which in turn reduced reactive oxygen species (ROS) production. Moreover, the activation of microglia and astrocytes was also found to decrease in the hippocampus. All of these changes greatly contributed to protect hippocampal neurons against ischemic insults.

**Conclusions**: PGC-1α could suppress the excessive ROS and neuroinflammation in the hippocampus, opening up a potential therapeutic target for cognitive impairment.

## Introduction

Vascular dementia (VaD), the second most common cause of dementia after Alzheimer's disease (AD), is characterized by memory and cognitive impairments 1. Many risk factors including an array of cardiovascular or cerebrovascular dysfunctions both lead to ischemic, cerebral hypoperfusion, or hemorrhagic brain lesions, which all contribute to the pathology of VaD 2. In addition, oxidative stress and neuroinflammation were also proved to play critical roles in the progression of VaD 3.

It was reported that glial activation could result in the white matter injury in mouse and rat models of chronic cerebral hypoperfusion 4-6. Moreover, activated microglia could also produce excessive reactive oxygen species (ROS), which in turn exacerbated neuroinflammation 3, 7. ROS mainly derives from dysfunctional mitochondria, and is extensively linked to neurodegenerative diseases, including AD and Parkinson's disease (PD) 8. As expected, suppressing ROS generation could significantly reduce hippocampal neuronal degeneration and attenuate cognitive impairment 9. Also, a previous study demonstrated that the repair for mitochondrial dysfunction could protect against hippocampal neuronal damage, thereby attenuating cognitive deficits 10. In the ischemic impaired hippocampus, the activities of oxidative phosphorylation complexes I, II, and IV were found to markedly decrease 11. All of these observations prompted us to hypothesize that an appropriate regulation for mitochondrial function might improve cognitive impairment.

Peroxisome proliferator-activated receptor-γ co-activator 1α (PGC-1α), a master regulator to regulate mitochondrial biogenesis 12, 13, mainly up-regulates mitochondrial DNA and induces the expressions of genes encoding mitochondrial proteins via interactions with estrogen-related receptor α or nuclear respiratory factor 1 and 2 14, 15. More specifically, PGC-1α powerfully promotes the expressions of ROS-detoxifying enzymes and uncoupling proteins (UCPs), thereby reducing ROS production 16. Importantly, the roles of PGC-1α in neurons have also been well studied. The overexpression of PGC-1α in dopaminergic neurons increased the expressions of mitochondrial antioxidants superoxide dismutase 2 (SOD2) and thioredoxin 2 (Trx2), and protected against the MPTP-induced cell degeneration in a mouse model of PD 17. In contrast, the reduced PGC-1α level, accompanied by the decreased expressions of mitochondrial antioxidants and UCPs, was correlated with the neuronal loss in multiple sclerosis (MS) patients 18. A previous study has reported that the expression of PGC-1α was also down-regulated in the brains of AD patients, which precisely reflects the extent of dementia 19. AD is characterized by the accumulation of Aβ and neuronal loss. PGC-1α was proved to block Aβ generation by modulating β-secretase, thereby reducing neuronal damage 20. Katsouri et al. also demonstrated that the up-regulated PGC-1α by lentivirus injection suppressed Aβ pathology and neuronal loss, and improved the cognitive function of APP23 transgenic mice, the mice of AD model 21. In addition, PGC-1α could also powerfully suppress the Aβ-induced neuroinflammation 21, 22. Thus, PGC-1α could act as an effective therapeutic target for cognitive impairment in AD.

To date, the role of neuronal PGC-1α in the progression of vascular cognitive impairment has not been investigated. In the present study, we speculate that neuronal PGC-1α promotes mitochondrial biogenesis, thereby reducing ROS production and improving the cognitive impairment induced by chronic cerebral hypoperfusion. To address this challenge, we specifically overexpressed PGC-1α gene in neurons using the Cre/LoxP system, which will contribute to verify the specific role of PGC-1α in VaD.

## Materials and methods

### Animals

Wild-type (WT) mice were purchased from the Vital River Corporation (Beijing, China). *PGC-1α*^f/f^ mice were generated by flanking Rosa26 of *PGC-1α* with *loxP* recombination sites, which contains a floxed *PGK*-neo-STOP cassette at the upstream of *PGC-1α*. The *PGC-1α*^f/f^ mice were generously provided by Zhenyu Ju. B6.Cg-Tg (Eno2-cre) 39Jme/J mice were purchased from Jackson Laboratory (stock number 006663). All mice were housed under specific pathogen-free conditions with a 12-h light/dark cycle, and given free access to food and water. All mice were C57BL/6 background. To generate mice that specifically overexpress PGC-1α in neurons, we crossed *PGC-1α*^f/f^ mice with Eno2-cre mice, and yielding offspring were bred with *PGC-1α*^f/f^ mice to generate *PGC-1α*^f/f^ Eno2-Cre (nPGC-1α) mice expressing IRES-eGFP, and the littermate control *PGC-1α*^f/f^ mice lacking the Cre recombinase transgene. The expressed Cre recombinase could excise the *PGK*-neo-STOP cassette, thereby inducing the neuronal specific overexpression of PGC-1α. The efficiency of PGC-1α overexpression was determined by Western blots.

A total of 262 male mice (12 weeks) were used in this present study, of which 95 were WT mice, 100 were* PGC-1α*^f/f^ mice and 67 were nPGC-1α mice. Mice were randomly assigned to different experimental groups by lottery drawing: WT group (n = 40), WT+bilateral common carotid artery stenosis (BCAS) group (n = 55), *PGC-1α*^f/f^ group (n = 55), *PGC-1α*^f/f^ +BCAS group (n = 45), nPGC-1α group (n = 27) and nPGC-1α+BCAS group (n = 40). All experimental protocols were approved by the Institutional Animal Care and Use Committee of Tianjin Medical University General Hospital.

### BCAS procedure

BCAS was used to induce chronic cerebral hypoperfusion, as previously described 4, 6, 23. Briefly, mice were anesthetized with chloral hydrate (30 mg/kg, intraperitoneally [ip]), and then the right common carotid artery was gently separated from the surrounding tissues and fully exposed from the carotid sheath. Next, the artery was wrapped with a microcoil (0.18-mm internal diameter). After 1 h, the same procedure was performed on the left common carotid artery. For the determination of PGC-1α level, WT mice were used as the sham control. The littermate *PGC-1α*^f/f^ mice were used as the sham control in the subsequent experiments, which underwent the same surgical procedure without the BCAS procedure. To clarify the role of PGC-1α, WT and nPGC-1α mice were treated with SR-18292, an indirect chemical inhibitor of PGC-1α, (45mg/kg, i.p. Sigma) for 4 weeks after BCAS surgery 24. Cerebral blood flow (CBF) was monitored by using a laser Doppler flowmetry at different time points, including before BCAS and at 2 h, 1 week, 2 weeks, and 4 weeks after BCAS surgery. These results were expressed as the percentages of the values measured before BCAS. Some mice died at some time during or after BCAS procedure, including 9 of 55 mice in the WT+BCAS group, 8 of 45 in the *PGC-1α*^f/f^ +BCAS group and 6 of 40 in the nPGC-1α+BCAS group. All data collected from died mice were excluded from analysis.

### In situ injection of lentivirus

To further investigate the role of PGC-1α, we knocked down and up-regulated the expression of PGC-1α in hippocampus by using the in situ injection of lentivirus. After anesthesia, WT mice were placed in a stereotaxic instrument. Lentivirus-PGC-1α, lentivirus-PGC-1α RNAi or lentivirus-control was injected into the hippocampus. The site of injection was located at the coordinates of 2.0 mm posterior to the bregma, 2.0 mm lateral to the midline, and 1.9 mm ventral to the skull surface. After 7 days, the BCAS surgery was performed on the mice with lentivirus injection.

### Morris water maze (MWM) experiment

At 4 weeks after surgery, spatial learning and memory abilities were tested using the MWM. The detailed procedures were carried out as previously described 25. Briefly, the maze consisted of a black plastic circular pool (120 cm diameter) filled with water to a depth of 45 cm. Water temperature was maintained at 23 ± 2°C during testing, and the water was made opaque using white ink. For data collection and analysis, the pool was divided into 4 virtual quadrants. An escape platform (10 cm diameter), invisible from the pool surface, was submerged 1 cm beneath the surface and placed at the center of one quadrant. This platform was consistently placed at the same place in the 'target' quadrant throughout the whole learning phase of behavioral training, and provided the only place of escape when a mouse was swimming in the water. At the place navigation stage, the mice were randomly place into the four quadrants and allowed to freely swim until they escaped to the platform. However, the mice were manually guided to the platform for 10 s if they failed to navigate to the platform within 2 min. A trial ended at this point or at the point when the mouse climbed onto the platform, which is the escape latency.

Each mouse underwent 4 trials per day for 5 consecutive days. The place navigation phase from day 1 to day 5 was used to evaluate the spatial learning ability. During this phase, we recorded escape latency and average swim speed. On day 6, a probe trial was conducted to evaluate the spatial memory ability. Before the trial, the platform was removed from the pool. Vorhees et al. suggested that the mice could be placed 180° from the original platform position 25, thus we chose the quadrant opposite to the target quadrant as a start position. The mouse was allowed to swim for a maximum of 2 min. The number of platform crossings and time spent in the target quadrant were calculated. The mice were monitored during learning and probe trials using a commercially available automated video system and Any-Maze software (Stoelting Co., Wood Dale, IL, USA).

### Odor discrimination reversal learning (ODRL)

ODRL test was performed to evaluate executive function, as previously reported 26, 27. In brief, the mice were tested in three stages, including shaping, acquisition and shift. Firstly, the mice in the testing chamber were trained to get reward food with lavender scent. This stage was not completed until these mice successfully acquired the food for 5 times. Secondly, this test proceeded to the stage of acquisition, and two cups of dried beans or wood chips were used in this test. Vanilla odor, mint odor and material pairings were randomly matched in each trail. Eight times out of ten in a trail was viewed to reach successful criteria. Lastly, these mice were immediately subjected to the shift stage. The cup with dried beans was defined as a bait. The successful criteria was the same as that of the acquisition stage. In the ODRL test, the trials to reach criteria and the time to find reward were used for the analysis for the executive function.

### Electrophysiological test

After the MWM test, mice were prepared for electrophysiological recording. Long-term potentiation (LTP) in the perforant pathway (PP) synapses of dentate gyrus (DG) was recorded to assess synaptic plasticity. LTP was viewed as a physical basis for memory encoding in the hippocampus 28. After anesthetization with 5% urethane (30 ml/kg, ip), the mice were placed in a small-rodent stereotaxic frame (Narishige, Japan). After exposing the skull surface, a small hole was made in the left dorsal skull using a dental drill, and a bipolar stimulation electrode was slowly lowered into the PP. Subsequently, a monopolar stainless steel electrode was lowered into the DG. The optimal coordinates of electrodes' locations were confirmed based on the previously reported data 29. Afterward, a stable normalized baseline was recorded for 20 min. Then theta burst stimulation (TBS) was used to induce LTP. After TBS, field excitatory postsynaptic potentials (fEPSP) were recorded every 60 s for 60 min (Scope Software, Powlab, AD Instruments, Australia). The mean fEPSP slope to reflect synaptic efficacy was normalized to the baseline.

### Cell culture and lentiviral transfection

HT-22 cells, an immortalized mouse hippocampal neuron cell line, were cultured in Eagle's Minimum Essential Medium (EMEM) (10-009-CVR, Corning) containing 10% fetal bovine serum (FBS, Life Technologies, Vienna, Austria) and 1% penicillin/streptomycin (Life Technologies) in 5% CO_2_ at 37°C. Before transfection, HT-22 cells were cultured in 24-well plates. Then, the PGC-1α-overexpressed lentivirus or negative control lentivirus (Genechem Co., Ltd., Shanghai, China) were transfected into HT-22 cells. The transfected cells were selected with puromycin (2 μg/mL; Santa Cruz) at 72 h after transfection. Transfection efficiency was evaluated by using Western blots.

### Oxygen and glucose deprivation (OGD) treatment

To generate a hypoxic environment for HT-22 cultures, we employed a self-contained and sealed hypoxia incubator chamber (Cat. 27310; STEMCELL Technologies Inc). After successful transfection, HT-22 cells were placed in this chamber (5% CO_2_ and 95% N_2_) and exposed to OGD treatment for 2 h in EMEM medium without serum and glucose. After OGD treatment, HT-22 cells were cultured in normal medium at 37°C for 24 h.

### High-throughput RNA sequencing (RNA-Seq)

Total RNA was extracted from the OGD-treated HT-22 cells using TRIzol reagent (Invitrogen, Carlsbad, CA, USA). Subsequently, the total RNA was identified and quantified by using NanoDrop ND-1000, and 1~2 μg of total RNA was used for the construction of RNA-Seq libraries. mRNA was enriched using NEBNext® Poly(A) mRNA Magnetic Isolation Module (New England Biolabs). Libraries were then constructed using KAPA Stranded RNA-Seq Library Prep kit (Illumina) according to the manufacturer's protocol. Sequencing library was determined by Agilent 2100 Bioanalyzer using the Agilent DNA 1000 chip kit (Agilent, part # 5067-1504). All samples were sequenced on Illumina HiSeq 4000 with 150 bp paired-end reads. After quality control, the raw sequencing data were aligned to the mouse genome (GRCm38) using Hisat2 software. Finally, differentially expressed genes were defined with fold change ≥ 1.5 and p value ≤ 0.05. Cluster analysis was performed using Cluster 3.0 software. Gene Ontology (GO) biological process analysis was performed using DAVID, and kyoto encyclopedia of genes and genomes (KEGG) was used for pathway analysis.

### Seahorse assay

The oxygen consumption rate (OCR) of HT-22 cells was determined by Seahorse Bioscience XF^e^96 Extracellular Flux Analyzer using XF Cell Mito Stress Test kit (Seahorse Bioscience, USA) according to the manufacturer's instructions. Briefly, HT-22 cells were seeded in Seahorse XF-96 plates at 10,000 cells/well, followed by OGD treatment for 2 h. After 24 h, these cells were used for Seahorse assay. Firstly, HT-22 cells were incubated in CO2-free incubator for 1 h before measurement. Secondly, the baseline of OCR was recorded. Then oligomycin (1 μM), FCCP (0.5 μM) and rotenone/antimycin A (1 μM) were subsequently added to reveal the key parameters showing metabolic function. Finally, basal respiration, ATP production, maximal respiration, spare capacity and proton leak were calculated.

### Hematoxylin and eosin (H&E) staining

After anesthetization, mice brains were gently removed and fixed in 4% paraformaldehyde overnight at 4°C. Brain tissues were then progressively dehydrated and embedded in paraffin. Coronal sections (8 μm) were cut using a microtome (Leica RM2255). Finally, these tissue sections were stained with an H&E kit (Solarbio, Beijing, China) according to the manufacturers' instructions.

### Microarray analysis

Total RNA was extracted from the hippocampus of nPGC-1α and *PGC-1α*^f/f^ mice by using TRIzol reagent according to the manufacturer's instructions. RNA was further purified to evaluate its concentration and integrity. Next, cDNA was generated and labeled with Cy3-dCTP fluorescent dye (GE Healthcare) using cRNA amplification and labeling kit (CapitalBio, Beijing, China). Then, denatured cDNA was loaded onto a Mouse Gene Expression 8 x 60k v2 microarray (Agilent Technologies), and hybridized in an Agilent Hybridization Oven overnight. Finally, the microarray was scanned using Agilent Microarray Scanner System (G2565CA). The raw data were extracted using Agilent Feature Extraction (v10.7) software, and further analyzed using GeneSpring software V13 (Agilent Technologies). GO and KEGG analyses were performed.

### Immunohistochemistry

After fixation in 4% paraformaldehyde, brain tissues were successively immersed in 15% and 30% sucrose for dehydration. After cutting, brain sections were incubated with 3% H_2_O_2_ for 10 min, followed by rinsing in PBS (3 × 5 min) to block endogenous peroxidase activity. After perforating with 0.2% Triton X-100 for 15 min, brain sections were blocked with 3% bovine serum albumin (BSA) for 30 min. Next, sections were incubated with the primary antibodies, including rabbit anti-brain-derived neurotrophic factor (BDNF) (1:500; Santa Cruz) and rabbit anti-neuron-specific DNA-binding protein (NeuN) (1:500; Abcam), overnight at 4°C. Subsequently, these sections were washed in PBS (3 × 10 min), followed by the incubation with horseradish peroxidase-labeled secondary antibody (1:500; DAKO, Glostrup, Denmark) for 1 h. Finally, the peroxidase reaction was developed with diaminobenzidine-tetrahydrochloride dihydrate (DAB; DAKO, Glostrup, Denmark) to make these sections visualized under a light microscopy.

Brain sections were fixed in cold acetone for 15 min, and incubated with 3% BSA, followed by the incubations with primary antibodies overnight at 4°C. The primary antibodies included rabbit anti-PGC-1α (1:50, Santa Cruz), rabbit anti-ionized calcium-binding adaptor molecule-1 (Iba-1) (1:500, Wako), rabbit anti-glial fibrillary acidic protein (GFAP) (1:1000, Abcam), rat anti-myelin basic protein (MBP) (1:1000, Abcam), mouse anti-CD68 (1:500, Abcam), rabbit anti-acetylcholinesterase (AChE) (1:50, Santa Cruz), rabbit anti-choline acetyltransferase (ChAT) (1:50, Santa Cruz), goat anti-vesicle acetylcholine transporter (VAChT) (1:50, Santa Cruz) and mouse anti-high-affinity choline transporter (ChT1) (1:50, Santa Cruz). Next, the sections were rinsed in PBS (5 × 5 min), followed by the incubations with the species-compatible fluorescent secondary antibodies for 1 h in the dark. After staining with Fluoroshield Mounting Medium with DAPI (Abcam, ab104139), these sections were observed under a fluorescent microscope.

Cell counting was carried out in a double-blind manner. Images for hippocampal layers were taken from 3 to 5 frozen sections from each mouse. The mean number of positively-stained cells in hippocampal CA1 area was quantified using ImageJ software, as previously described 18. Data were expressed as the mean number of positively stained-cells per visual field.

### Quantitative real-time PCR (qRT-PCR)

Total RNA was extracted from fresh hippocampal tissues by using TRIzol reagent. Subsequently, cDNA was synthesized using TransScript First-Strand cDNA Synthesis Super Mix (TransGen Biotech, China). Next, qPCR reactions were performed on a CFX Connect^TM^ Real-Time PCR Detection System (Bio-Rad, USA) using FastStart Universal SYBR Green Master (Roche, Germany) in triplicate. The housekeeping gene GAPDH was used as a normalized control. The following PCR conditions were used: denaturation at 95°C for 10 min, followed by 39 cycles of 95°C for 15 s and 60°C for 1 min. The relative mRNA expression was calculated using the 2^-ΔΔCt^ comparative method. All primers for amplification were shown in Table S1.

### Western blots

Hippocampal tissues were homogenized by sonication in 200 μL RIPA buffer containing a protease inhibitor cocktail. The concentration of proteins was determined using a BCA kit. Equal amount of proteins were separated using SDS-PAGE gels, and then the separated proteins were transferred onto PVDF membranes (Millipore, USA) using the semi-dry transfer method. After blocking with 5% nonfat dried milk for 2 h, the membranes were incubated with primary antibodies overnight at 4°C. The primary antibodies included rabbit anti-PGC-1α (1:1000, ThermoFisher), rabbit anti-BDNF (1:200, Santa Cruz), mouse anti-AChE (1:100, Santa Cruz), rabbit anti-ChT1 (1:1000, Abcam), rabbit anti-ChAT (1:1000, Abcam) and goat anti-VAChT (1:1000, Santa Cruz) antibodies. Next, the membranes were incubated with the species-compatible secondary antibodies for 1 h. The specific protein bands were detected using a Bio-Rad Gel Doc Imager.

### Detection of ROS production in situ

Dihydroethidium (DHE, Sigma-Aldrich) was used to detect the in situ ROS production in hippocampus, in which DHE could be oxidized to red fluorescent molecule ethidium by superoxide. Mice were injected (ip) with DHE (100 μL, 4 mg/mL) 3 times at 1-h intervals. Then, the brain tissues were removed and cut. Finally, brain sections were incubated with DAPI and observed under a fluorescent microscope. And the DHE fluorescence intensity was quantified using ImageJ software.

### Statistical analysis

All analyses were carried out using GraphPad Prism 5.0 software to evaluate the statistical differences among groups. Data were presented as means ± SEM. Two-way analysis of variance (ANOVA) was used to compare the time-course data, including escape latencies, mean swimming speed and CBF. One-way ANOVA (Kruskal-Wallis test) followed by an appropriate *post-hoc* test was used to test the differences among multiple groups. The Mann-Whitney *U* test was used to compare the differences between two groups. Differences were considered significant at p < 0.05.

### Data deposition

RNA-Seq and microarray data were deposited at the Gene Expression Omnibus (GEO) database (http://www.ncbi.nlm.nih.gov/geo/) with accession numbers GSE127788 and GSE134257, respectively.

## Results

### Decreased expression of hippocampal PGC-1α following chronic cerebral hypoperfusion

**Figure 1:**
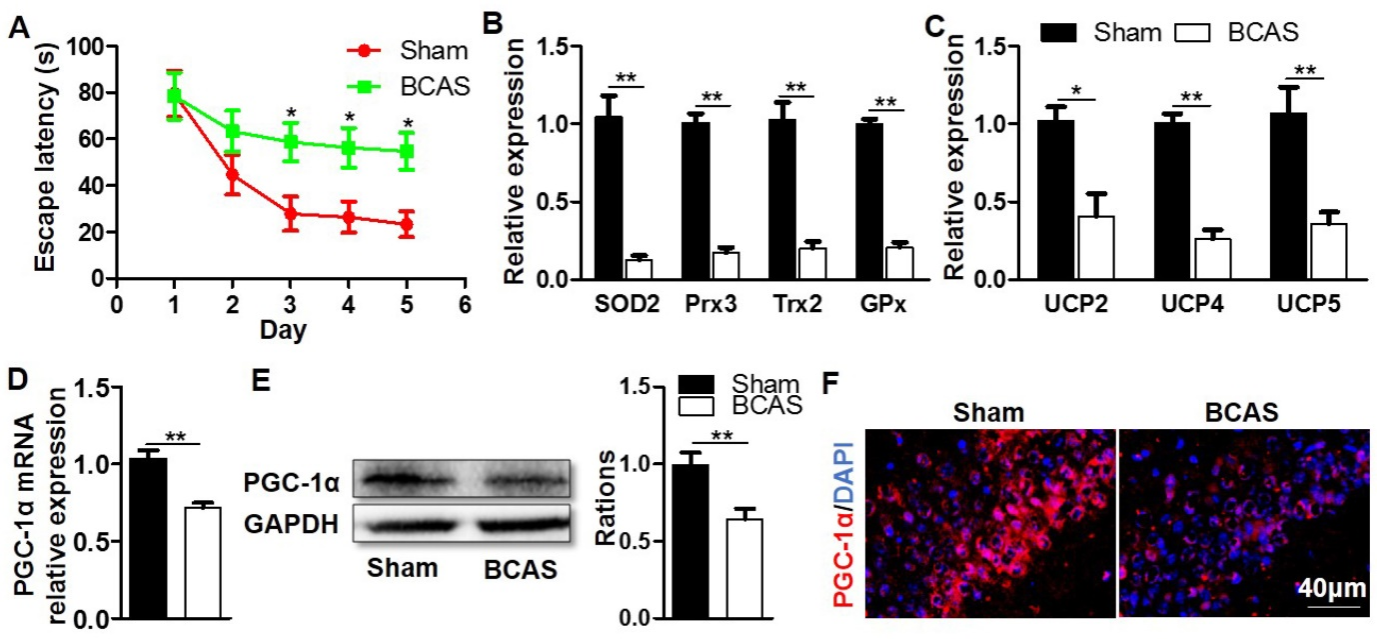
** Decreased expression of hippocampal PGC-1α in the mice with chronic cerebral hypoperfusion.** Wild-type mice were used to establish the VaD model with the chronic cerebral hypoperfusion induced by BCAS. (**A**) Evaluation of learning ability for BCAS and sham mice using MWM test. Mean escape latency was longer in the BCAS group at the place navigation stage, revealing the impaired spatial learning ability. (**B**) qRT-PCR analysis showed a significant reduction in the mRNA expressions of mitochondrial antioxidants in the hippocampus of BCAS group compared to the sham group. (**C**) The mRNA expressions of hippocampal UCPs were also significantly down-regulated in the BCAS group. The levels of hippocampal PGC-1α mRNA (**D**) and protein (**E**) expressions were both significantly down-regulated in the BCAS group. (**F**) Representative images of immunofluorescent staining clearly showed the decreased PGC-1α expressions in the hippocampal CA1 areas of BCAS mice. *p<0.05, **p<0.01 as determined by two-way ANOVA (**A**) or Mann-Whitney *U* test (**B-E**). n = 6 in each group.

To investigate the possibility that PGC-1α may be related to cognitive impairment, we firstly established a mouse model in which the cognitive impairment was induced by chronic cerebral hypoperfusion. MWM experiment indicated that the mice from BCAS group had a longer escape latency to find the hidden platform compared with those from the sham group on days 3 to 5 at the place navigation stage, suggesting that they had impaired spatial learning ability and cognitive impairment (Figure 1A). After the MWM test, these mice were immediately sacrificed to collect their hippocampal tissues, then the key mitochondrial antioxidants and UCPs in the hippocampus were measured. The results showed that the mRNA expressions of SOD2, peroxiredoxin 3 (Prx3), Trx2, and glutathione peroxidase 1 (GPx1) were all significantly down-regulated in the BCAS group (Figure 1B), and the mRNA expressions of UCP2, UCP4 and UCP5 were also greatly down-regulated (Figure 1C). Given that PGC-1α was involved in regulating the transcriptions of mitochondrial antioxidants and UCPs 16, we also analyzed the expression of PGC-1α in the hippocampus. The levels of PGC-1α mRNA and protein expressions markedly decreased in the BCAS group relative to that in the sham group (Figure 1D-E). The hippocampal CA1 area was proved to be more closely related to the cognitive dysfunction 30, 31. However, the immunofluorescent staining confirmed that PGC-1α expression was down-regulated in the hippocampus CA1 area of mice from the BCAS group (Figure 1F). Additionally, the expression of PGC-1α was also down-regulated in the cortex and hippocampal CA3 area of mice from the BCAS group compared to that from the sham group (Figure S1). In summary, we observed a significant reduction in PGC-1α level in the hippocampus of mice with cognitive deficits, which is likely to underlie the decreased expressions of mitochondrial antioxidant enzymes and UCPs.

### Characteristics of transgenic mice overexpressing neuron-specific PGC-1α

**Figure 2:**
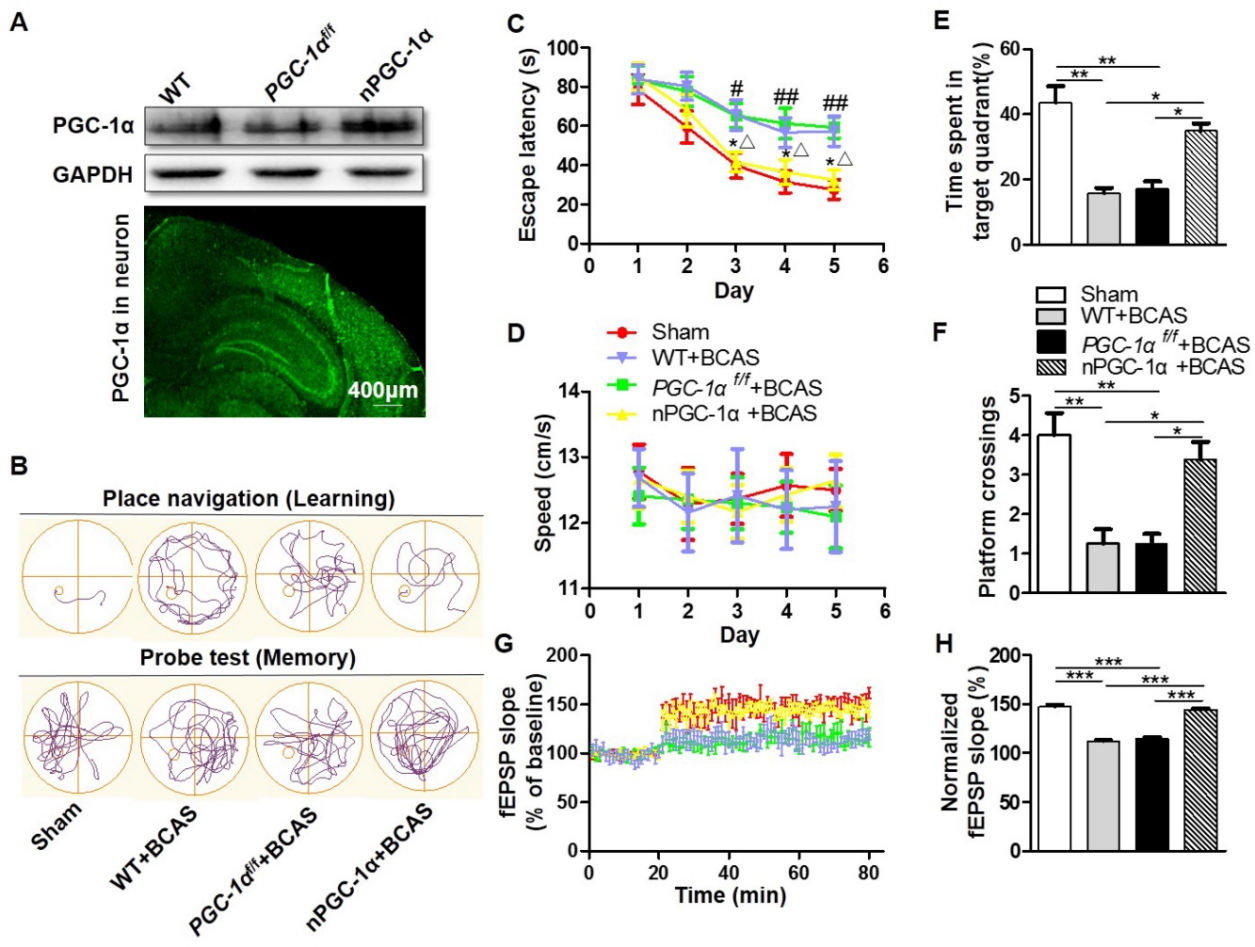
** PGC-1α protects against the cognitive impairment caused by chronic cerebral hypoperfusion.** (**A**) Top, Western blots for PGC-1α expressions in the hippocampus from WT, *PGC-1α^f/f^*and nPGC-1α mice. Bottom, a representative image for the coronal brain section prepared from nPGC-1α mice showed the neuronal expression of IRES-eGFP. (**B**) The typical swimming paths of sham-operated, WT+BCAS, *PGC-1α^f/f^* +BCAS, and nPGC-1α+BCAS mice in the MWM during learning (top) and memory probe tests (bottom). (**C**) Escape latencies were much longer in WT+BCAS and *PGC-1α^f/f^* +BCAS mice than those in the sham mice or nPGC-1α+BCAS mice. *p<0.05 for the comparison between *PGC-1α^f/f^* +BCAS and nPGC-1α+BCAS groups; ^#^p<0.05, ^##^p<0.01 for the comparison between the sham and *PGC-1α^f/f^ +*BCAS groups; ^△^p<0.05 for the comparison between WT+BCAS and nPGC-1α+BCAS groups, as determined by two-way ANOVA. (**D**) Mean swimming speed of these 4 groups of mice during spatial training was similar, showing that swim speed may not contribute to the differences in escape latencies. Mean percentage of time spent in target training quadrant (**E**), and mean number of platform crossings (**F**) during the probe test. (**G**) fEPSPs slopes were continuously recorded. (**H**) Mean fEPSP slope of LTP between 40 min and 60 min after TBS. *p<0.05, **p<0.01, ***p<0.001 as determined by one-way ANOVA. (**A, G-H**) n = 5 in each group, (**B-F**) n = 8 in each group.

Considering that the dysfunction of hippocampal neurons greatly contributes to the cognitive impairment in AD 32, PGC-1α level was determined and found to be significantly changed following BCAS in our study. Then, the transgenic mice that overexpress neuron-specific PGC-1α were generated via the Cre/LoxP system to evaluate the neurological function after chronic cerebral hypoperfusion. To test the efficiency of PGC-1α overexpression, we quantified PGC-1α expression by using Western blots. The PGC-1α expression was up-regulated in nPGC-1α mice (Figure 2A). In addition, the IRES-eGFP-positive neurons in frozen brain sections were observed using fluorescence microscopy, demonstrating that PGC-1α was overexpressed in these neurons (Figure 2A). Further experiments showed that the brain structure and neuronal density in cortex and hippocampal CA1 area were not obviously altered in WT, *PGC-1α^f/f^* or nPGC-1α mice, suggesting that the overexpression of neuronal PGC-1α may not alter the brain environment under normal physiological condition (Figure S2). CBF was also recorded for assessment of the blood flow before and after BCAS surgery, and the results indicated that from hours to weeks after BCAS, the CBF significantly decreased in those BCAS groups compared to that in the sham group. However, CBF was comparable among three BCAS groups, suggesting that the variability in genotypes is not attributable to the difference in blood flow (Figure S3).

### PGC-1α improves cognitive performance in mice after chronic cerebral hypoperfusion

The MWM was performed for the neurological evaluation of spatial learning and memory abilities in the neuronal PGC-1α-overexpressed mice after chronic cerebral hypoperfusion. The typical swimming pathways of mice from the sham, WT+BCAS, *PGC-1α^f/f^*+BCAS, and nPGC-1α +BCAS groups were shown in Figure 2B. The results showed that mean escape latency was longer in the WT+BCAS and *PGC-1α^f/f^* +BCAS groups compared to that in the sham and nPGC-1α+BCAS groups, indicating that neuronal PGC-1α overexpression significantly improved the learning ability (Figure 2C). In addition, the* PGC-1α^f/f^* +BCAS group had a longer latency time compared to the sham and nPGC-1α+BCAS groups. The WT+BCAS group performed similarly to the* PGC-1α^f/f^* +BCAS group, and no significant difference was observed in the performance between the sham and nPGC-1α+BCAS groups (Figure 2C). All these 4 groups exhibited similar swimming speeds, suggesting that the mice had comparable swimming skills (Figure 2D), ruling out potential confounding factors to obtain better performance. In the spatial probe trial, the mice from the WT+BCAS and *PGC-1α^f/f^* +BCAS groups spent less time in the target quadrant and had fewer platform crossings than that from the sham group. Interestingly, the above parameters were improved in the nPGC-1α+BCAS group (Figure 2E-F). Using ODRL test, we found that the mice from WT+BCAS and *PGC-1α^f/f^* +BCAS groups had executive function deficits, while PGC-1α overexpression improved executive function of mice from the nPGC-1α+BCAS group (Figure S4). To further confirm the neuroprotective effects of PGC-1α, the in situ injection of lentivirus was performed to regulate the level of PGC-1α in hippocampus. As expected, the knockdown of PGC-1α worsened the cognitive impairment compared with the control, but the up-regulation of PGC-1α improved the abilities of learning and memory (Figure S5A-C). In addition, the neuroprotective effect of PGC-1α on the nPGC-1α+BCAS group was reversed by SR-18292, an inhibitor of PGC-1α. In the WT+BCAS group, SR-18292 greatly aggravated the neurological outcomes (Figure S5D-E). Taken together, these results suggested that the PGC-1α overexpression in neurons could improve hippocampal neuronal function and attenuate cognitive impairment after chronic cerebral hypoperfusion.

After the MWM test, we used LTP to assess hippocampal synaptic plasticity, which is necessary for assessing the learning and memory functions. Basal perforant-path evoked fEPSPs in DG of hippocampal formation were recorded. In this assessment, LTP was induced by TBS, and the evoked fEPSPs slopes in DG were recorded for 1 h and the amplitudes were normalized to the 20 min baseline period. The results showed that the mean fEPSP slope significantly decreased in WT+BCAS and* PGC-1α^f/f^* +BCAS groups compared to the sham and nPGC-1α+BCAS groups. However, no significant difference was observed between the sham and nPGC-1α+BCAS groups (Figure 2G-H). These data suggest that the neuronal PGC-1α also promotes the recovery of cognitive function.

### PGC-1α overexpression alters the gene expression profile of neurons

**Figure 3:**
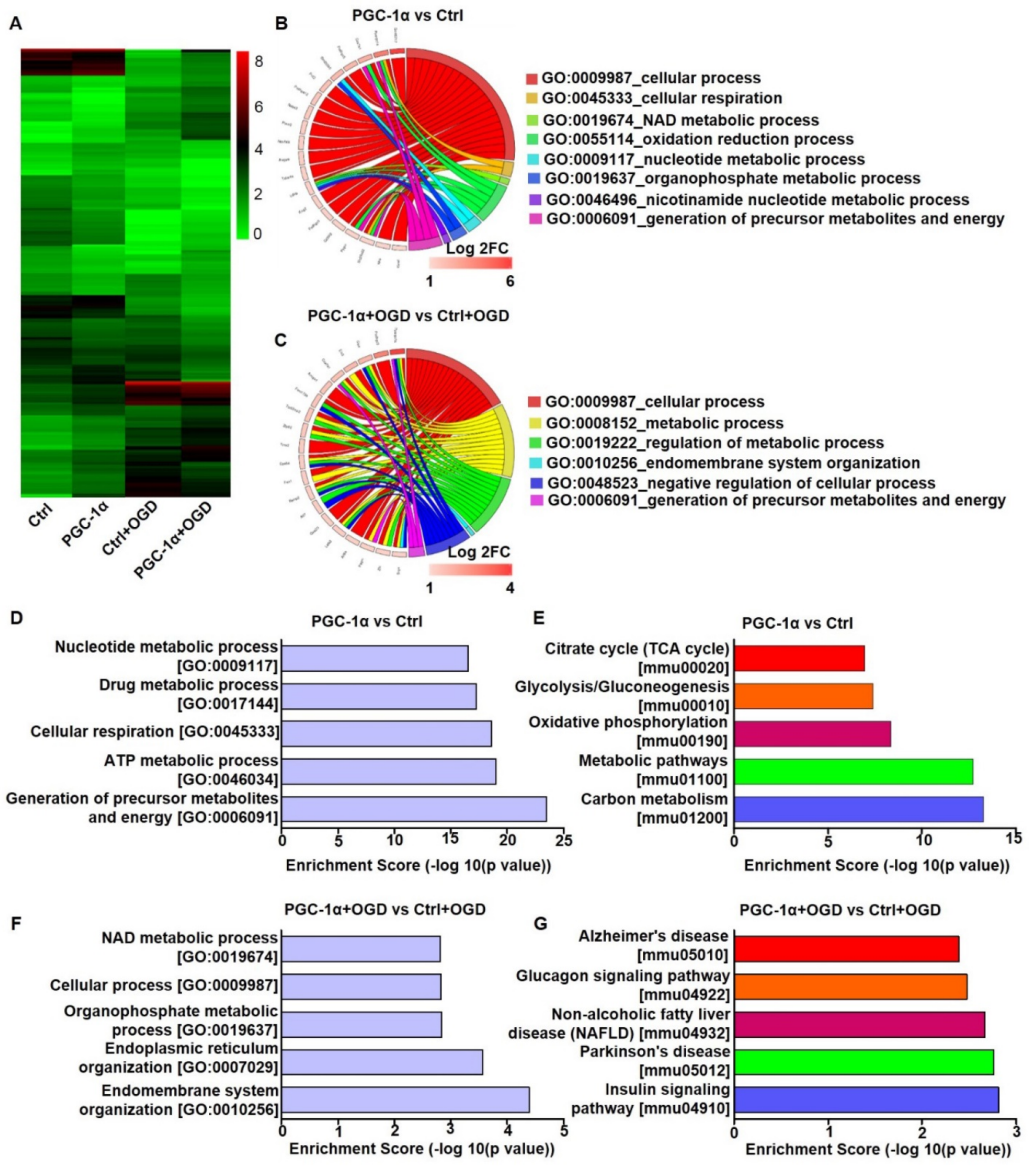
** PGC-1α overexpression alters the gene expression profile of cultured neurons.** (**A**) Cluster analysis for the differentially expressed mRNAs of control, PGC-1α, control+OGD and PGC-1α+OGD groups based on RNA-Seq data. Circle plot showed that the top 20 up-regulated genes were closely related to several biological processes in PGC-1α vs control groups (**B**) and PGC-1α+OGD vs control+OGD groups (**C**). (**D**) Go enrichment analysis for the differentially expressed genes that were up-regulated by PGC-1α. The top 5 enriched biological processes were shown. (**E**) KEGG pathway analysis for the PGC-1α-upregulated genes after OGD treatment. The top 5 pathways were shown. (**F**) The bar graph showed the top 5 up-regulated biological processes in the PGC-1α+OGD group compared to the control+OGD group. (**G**) The top 5 pathways that were up-regulated in the PGC-1α+OGD group compared to the control+OGD group.

To investigate the neuroprotective mechanism of PGC-1α, we performed RNA-Seq analysis on the PGC-1α-overexpressed HT-22 cells, a hippocampal neuron cell line. In this analysis, the cells received OGD to mimic chronic cerebral hypoperfusion. RNA-Seq analysis showed that 336 genes were up-regulated, and 363 genes were down-regulated in PGC-1α-overexpressed neurons compared to controls. Furthermore, PGC-1α increased the expressions of 170 genes, and reduced the expressions of 171 genes after OGD treatment (Figure 3A). GO analysis for the top 20 PGC-1α-upregulated genes under normal condition showed that these genes were closely related to several biological processes, like cellular respiration, NAD metabolic process, oxidation reduction process and the generations of precursor metabolites and energy (Figure 3B). After OGD treatment, the up-regulated genes by PGC-1α were also associated with biological processes, including metabolic process and the generations of precursor metabolites and energy (Figure 3C). These results indicated that PGC-1α participated in regulating the energy metabolism of neurons. GO analysis for the 336 PGC-1α-upregulated genes revealed that these genes were involved in the generations of precursor metabolites and energy, ATP metabolic process, cellular respiration, drug metabolic process and nucleotide metabolic process (Figure 3D). KEGG pathway analysis for these 336 genes indicated that these genes were involved in carbon metabolism, metabolic pathways, oxidative phosphorylation, and glycolysis/gluconeogenesis and citrate cycle (TCA cycle) (Figure 3E). These findings indicated that neuronal PGC-1α could promote generating a more favorable energy environment before hypoperfusion, which may in turn protect against neuronal damage. After OGD treatment, GO analysis for the 170 up-regulated genes by PGC-1α showed that the top 5 biological processes included endomembrane system organization, endoplasmic reticulum organization, organophosphate metabolic process, cellular process and NAD metabolic process (Figure 3F). KEGG pathway analysis for these genes identified several key signaling pathways, including the insulin signaling pathway, Parkinson's disease, non-alcoholic fatty liver disease (NAFLD), glucagon signaling pathway, and Alzheimer's disease (Figure 3G). Taken together, the RNA-Seq data indicated that PGC-1α might enhance metabolic processes of neurons, thereby protecting against cognitive deficits after ischemia insult.

In basal conditions, the gene expression profiles of hippocampus between *PGC-1α^f/f^* and nPGC-1α mice were measured by using microarray analysis. We found that a total of 100 genes were up-regulated and 290 genes were down-regulated in the hippocampus from nPGC-1α mice relative to *PGC-1α^f/f^* mice (Figure S6A). GO analysis for these differentially expressed genes indicated that the molecular function for these PGC-1α-induced genes mainly focused on the two aspects of energy metabolism and inflammation, such as hemoglobin binding, CCR chemokine receptor binding, chemokine activity, phospholipid-translocating ATPase activity and maltose alpha-glucosidase activity (Figure S6B). KEGG analysis also showed the corresponding enriched pathways, including ubiquitin mediated proteolysis, galactose metabolism, cytokine-cytokine receptor interaction, glycolysis/gluconeogenesis, inflammatory mediator regulations of TRP channels and NF-kappa B signaling pathway (Figure S6C). Altogether, these results indicated that PGC-1α regulated the energy metabolism and inflammation in the hippocampus.

### PGC-1α increases the mitochondrial respiration in neurons

**Figure 4:**
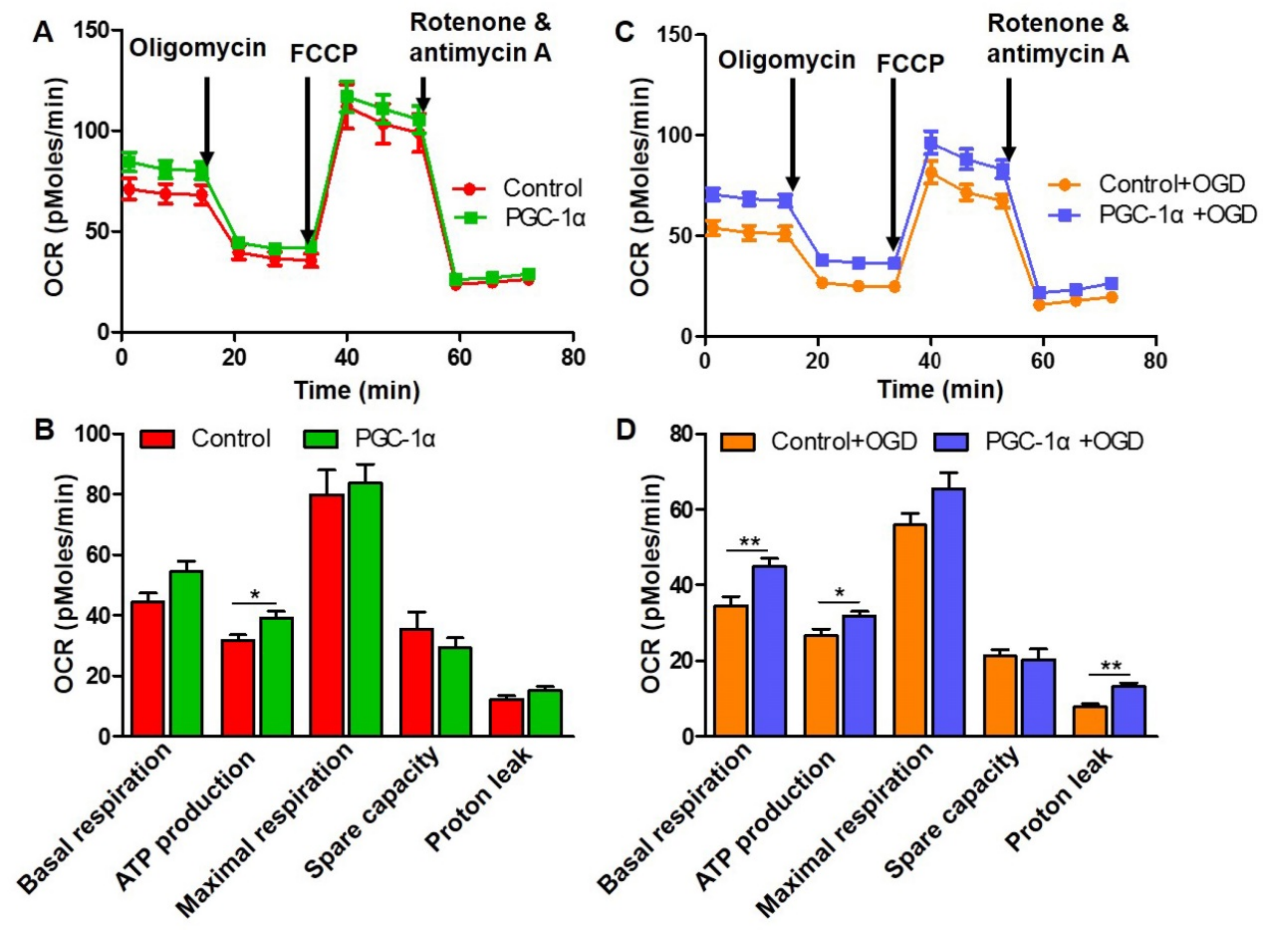
** PGC-1α increases the mitochondrial respiration in neurons.** (**A**) Seahorse assay for the OCR in HT-22 cells with or without PGC-1α overexpression. (**B**) Quantification of basal respiration, ATP production, maximal respiration, spare capacity and proton leak. PGC-1α increased the ATP production of neurons. (**C**) OCR was measured in HT-22 cells after OGD treatment. (**D**) The 5 key parameters for mitochondrial respiration were calculated from OCR. *p<0.05, **p<0.01 as determined by Mann-Whitney *U* test. n = 8 in each group.

To further confirm whether PGC-1α increases the metabolic activity of neurons, we assessed mitochondrial respiration by measuring OCR in HT-22 cells using Seahorse assay. We found that PGC-1α increased the ATP production of neurons (Figure 4A-B), which suggests that PGC-1α overexpression can better meet the energetic needs of neurons under the basal condition. In addition, there were no differences in the basal respiration, maximal respiration, spare capacity and proton leak between the PGC-1α-overexpressed HT-22 cells and control HT-22 cells (Figure 4A-B). After OGD treatment, we also found that the basal respiration, ATP production and proton leak significantly increased in PGC-1α-overexpressed HT-22 cells compared to the HT-22 cells without PGC-1α overexpression (Figure 4C-D). Altogether, these data indicate that PGC-1α could enhance the energetic metabolism of neurons, which in turn generally improves the neuronal function.

### PGC-1α induces hippocampal BDNF expression after chronic cerebral hypoperfusion

**Figure 5:**
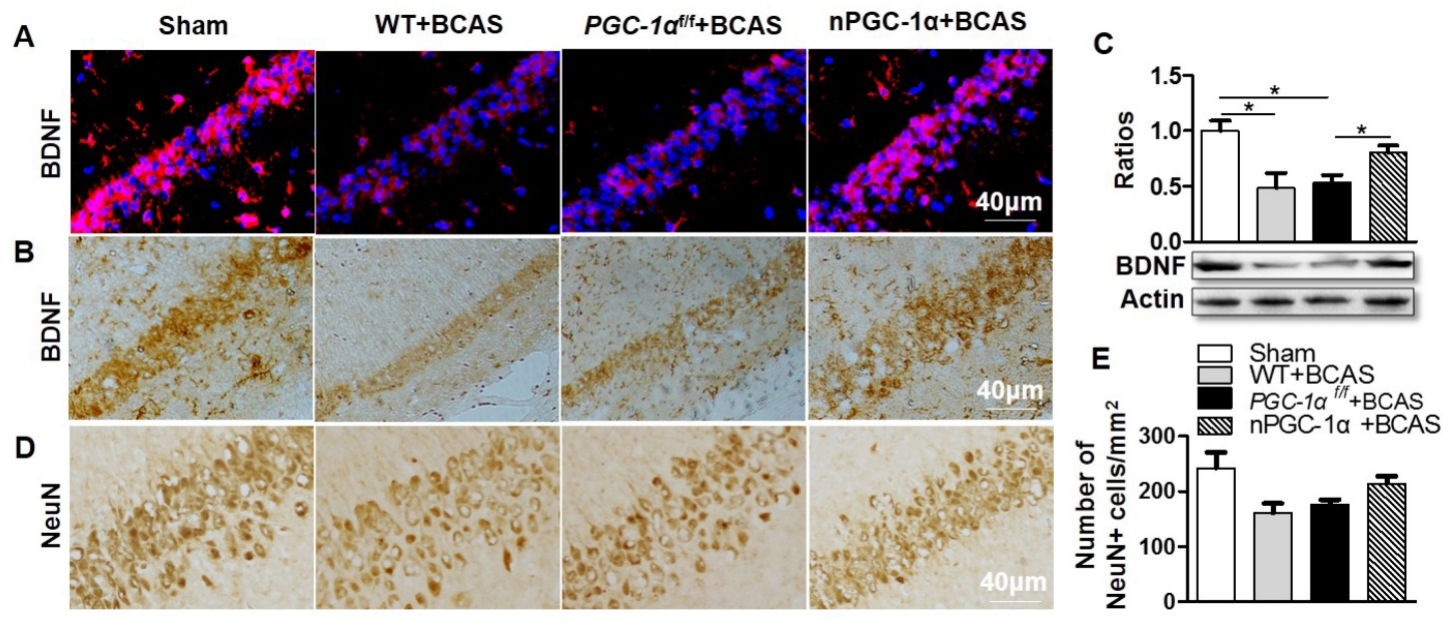
** PGC-1α induces hippocampal BDNF expression after chronic cerebral hypoperfusion.** Representative images of brain sections immunostained for BDNF (**A, B**), and Western blots for BDNF (**C**) showed that BDNF protein was significantly downregulated in WT+BCAS and *PGC-1α^f/f^* +BCAS groups compared to the sham group. By contrast, BDNF was up-regulated in the nPGC-1α+BCAS group. (**D, E**) Immunostaining in hippocampal CA1 area showed that there was only a downward trend for the numbers of NeuN-positive neurons in the WT+BCAS and *PGC-1α^f/f^* +BCAS groups compared to the sham and nPGC-1α+BCAS groups. *p<0.05 as determined by one-way ANOVA. n = 5 in each group.

To further investigate the neuroprotective mechanism of PGC-1α, the level of BDNF was evaluated, which was proved to be essential for assessing the synaptic plasticity, normal hippocampal function and neuron survival 33. Immunohistochemical and Western blot analyses revealed a significant reduction in BDNF expressions in the hippocampal CA1 area of WT+BCAS and* PGC-1α^f/f^* +BCAS mice compared to sham mice. In contrast, BDNF levels were up-regulated in the nPGC-1α+BCAS group (Figure 5A-C). These results strongly supported the hypothesis that the neuronal PGC-1α enhances BDNF expression. Furthermore, the numbers of neurons in the hippocampal CA1 area of the mice were also evaluated. The results indicated that no significant differences among these four groups were observed, but there appeared to be fewer neurons in the hippocampal CA1 area of WT+BCAS and* PGC-1α^f/f^* +BCAS groups compared to the sham group, and the numbers of neurons in nPGC-1α+BCAS group tended to increase (Figure 5D-E). These results suggest that PGC-1α promotes BDNF expression, which may potentially contribute to the neuronal restoration and repair at the chronic recovery stage following BCAS.

### Cholinergic dysfunction is attenuated in the mice overexpressing neuronal PGC-1α

**Figure 6:**
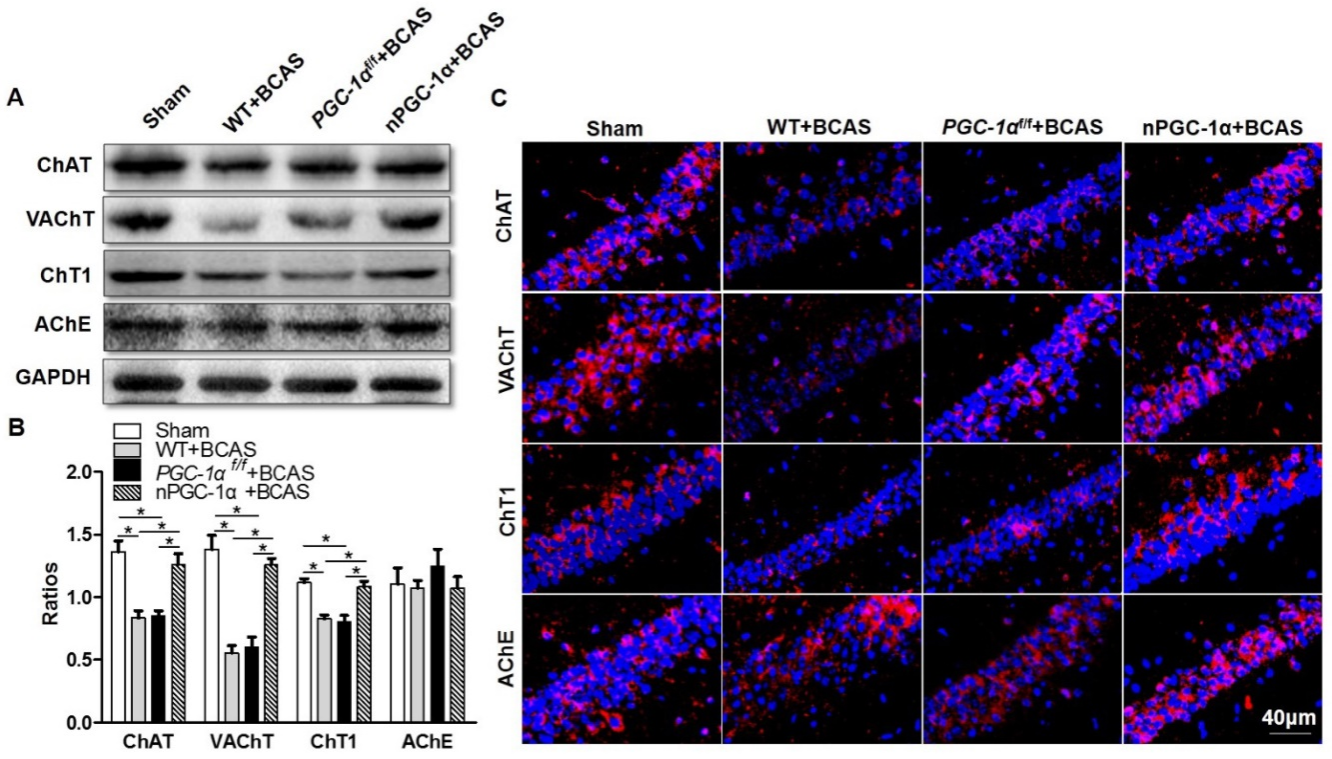
** Neuronal PGC-1α improves the cholinergic dysfunction in the hippocampus.** (**A, B**) Representative images of Western blots demonstrated the decreased ChAT, VAChT and ChT1 in the hippocampus of mice from WT+BCAS and* PGC-1α^f/f^* +BCAS groups compared to the sham and nPGC-1α+BCAS groups. There were no significant differences in AChE expression among 4 groups. (**C**) Representative images of cholinergic immunofluorescence staining showed a similar trend. *p<0.05 as determined by one-way ANOVA. n = 6 in each group.

Disruption in the central cholinergic neurotransmitter system has been observed in AD and VaD, and this disruption could greatly contribute to cognitive deficits 34, 35. Moreover, cerebral hypoperfusion was proved to lead to cholinergic deficits, eventually aggravating cognitive impairment 36. A previous study indicated that cholinergic neurotransmitter mainly originated from neurons 37, and the hippocampus had a higher density of cholinergic innervation 38. Thus, we further determined the cholinergic activity in the hippocampal CA1 area of the PGC-1α-overexpressed mice. We quantified the levels of cholinergic components, including ChAT, VAChT, ChT1 and AChE. The results showed that the expression of ChAT, a direct synthetase for acetylcholine (ACh), markedly decreased in the hippocampal CA1 area of the mice from WT+BCAS and *PGC-1α^f/f^* +BCAS groups compared to that from the sham group. By contrast, this decrease was improved in the nPGC-1α+BCAS group (Figure 6A-B). In addition, the comparable changes were observed in VAChT and ChT1 levels. In general, VAChT is involved in the storage and transportation of ACh, while ChT1 is the rate-limited enzyme to synthesize ACh. At last, no significant difference in the expression of AChE, a degradation enzyme for ACh, was observed among these 4 groups (Figure 6A, C). These results together suggest that the neuronal PGC-1α regulates the cholinergic activity of neurons, further improving the learning and memory functions.

### PGC-1α reduces oxidative stress after chronic cerebral hypoperfusion

**Figure 7:**
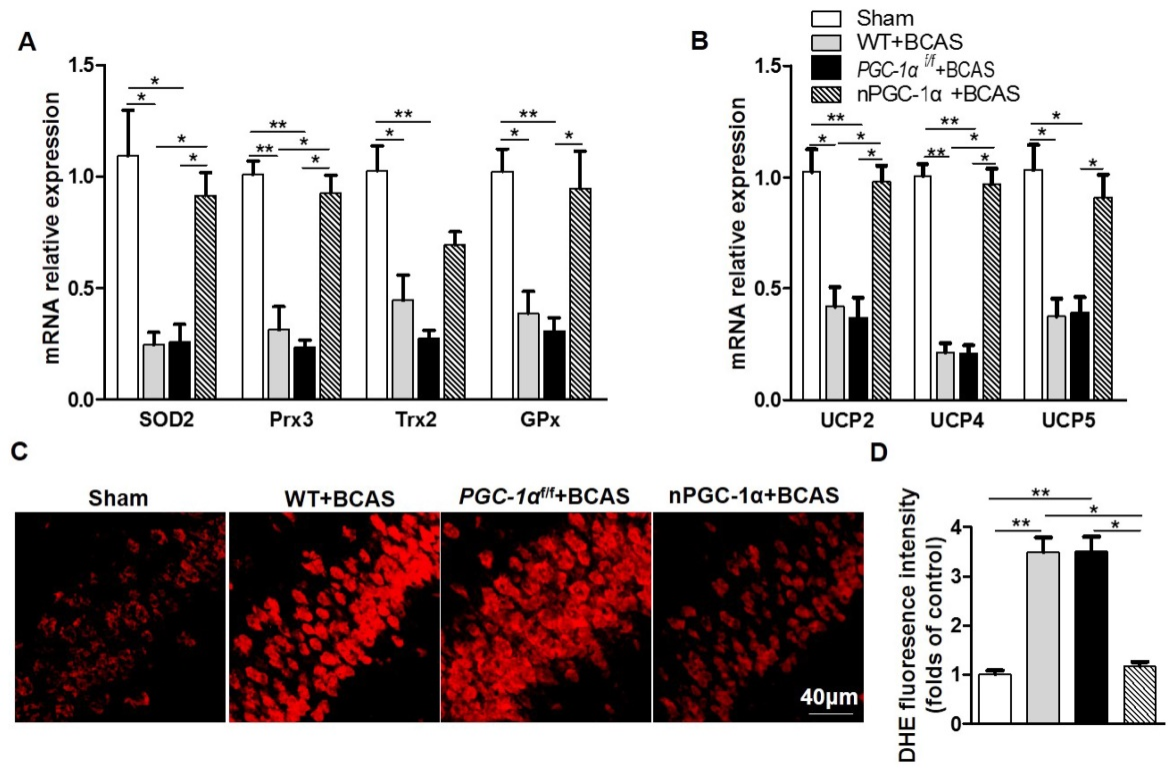
** PGC-1α reduces ROS production.** (**A**) The relative mRNA expressions of mitochondrial antioxidants including SOD2, Prx3, Trx2 and GPx1 were determined by qRT-PCR. (**B**) The relative mRNA expressions of UCPs including UCP2, UCP4 and UCP5 in the hippocampus from 4 groups were also measured. (**C**) Representative images of DHE staining reflected the ROS levels in the hippocampus of these 4 groups, indicating that PGC-1α greatly reduced ROS production. (**D**) DHE fluorescence intensity was quantified, and then expressed as the folds of control. *p<0.05, **p<0.01 as determined by one-way ANOVA. n = 6 in each group.

PGC-1α has been shown to modulate the metabolic activity of neurons, which substantially improved BDNF production, cholinergic activity and learning ability after chronic cerebral hypoperfusion. However, the underlying neuroprotective mechanism of PGC-1α is unclear. Therefore, we further attempted to unravel the role of PGC-1α in the transcriptional regulations of mitochondrial antioxidants and UCPs under chronic cerebral hypoperfusion condition. It has been verified that mitochondrial antioxidants, such as SOD2, Prx3, Trx2, and GPx1, could exert neuroprotective effects by eliminating ROS 39, 40. In addition, UCPs could also reduce ROS production via modulating the mitochondrial electron transport chain. We found that the mRNA expressions of SOD2, Prx3, and GPx1 significantly decreased in the hippocampus of the mice from WT+BCAS and *PGC-1α^f/f^* +BCAS groups compared to the sham group, while this decrease was reversed in the nPGC-1α+BCAS group (Figure 7A). In addition, the mRNA expressions of UCP2, UCP4 and UCP5 also reduced in the hippocampus of the mice from WT+BCAS and *PGC-1α^f/f^* +BCAS groups compared to the sham group, and their expressions were increased in the nPGC-1α+BCAS group (Figure 7B). We also measured the level of ROS in the hippocampus using DHE staining, and found that the ROS levels obviously increased in WT+BCAS and *PGC-1α^f/f^* +BCAS groups compared to the sham group. However, the mice overexpressing neuronal PGC-1α had a lower ROS level (Figure 7C-D). These results suggest that one possible mechanism for the neuroprotective effects of PGC-1α is that it greatly reduces ROS production in mice after chronic cerebral hypoperfusion.

### Glial activation is attenuated in the mice overexpressing neuronal PGC-1α

**Figure 8:**
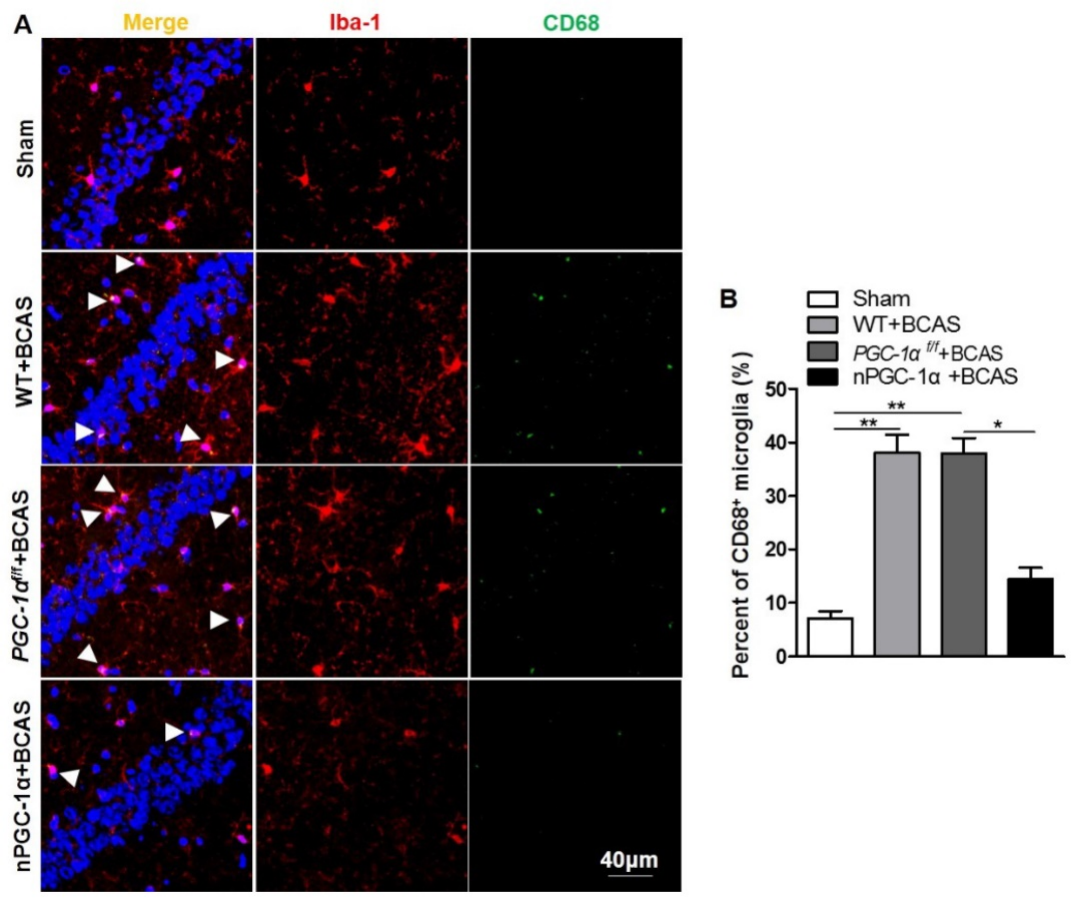
** PGC-1α attenuates the activation of microglia in hippocampus.** (**A**) Immunofluorescent staining of the CD68-positive microglia in the hippocampal CA1 areas of the WT+BCAS, *PGC-1α^f/f^* +BCAS, nPGC-1α+BCAS and sham mice after chronic cerebral hypoperfusion. (**B**) The percent of the CD68-positive microglia. *p<0.05, **p<0.01 as determined by one-way ANOVA. n = 6 in each group.

Previous studies indicated that cerebral hypoperfusion could induce glial activation and neuroinflammation 4, 5, which plays crucial roles in the pathophysiology of VaD 41. CD68, an activated microglia marker, was evaluated in our study 42, 43. We observed that the percent of CD68-positive microglia increased in the hippocampus CA1 area of the mice from WT+BCAS and *PGC-1α^f/f^* +BCAS groups compared to the sham group, while the overexpression of PGC-1α reduced the CD68-positive microglia after chronic cerebral hypoperfusion (Figure 8A-B), decreasing the activation of microglia. In addition, the activation of astrocytes significantly increased in WT+BCAS and *PGC-1α^f/f^* +BCAS groups compared to the sham group, but decreased in the nPGC-1α+BCAS group compared to WT+BCAS and *PGC-1α^f/f^* +BCAS groups (Figure S7A-B). Furthermore, the reduced damage of white matter and the improved repair for myelin sheath were observed in the nPGC-1α group (Figure S8). Altogether, the neuronal PGC-1α-induced cognitive improvement was accompanied with the decreased neuroinflammation. Thus, the low intensity of inflammation may promote the neuroprotective effects of neuronal PGC-1α.

## Discussion

In this present study, we observed that PGC-1α expression is markedly down-regulated in the hippocampus of mice with cognitive impairment induced by chronic cerebral hypoperfusion. To determine the role of PGC-1α in neurons under ischemic condition, we generated a strain of transgenic mouse that overexpresses the neuron-specific PGC-1α. We found that PGC-1α could improve the learning, memory and executive functions in the mice subjected to chronic cerebral hypoperfusion. We also observed that PGC-1α reduced ROS production. In addition, PGC-1α overexpression reduced the glial activation in hippocampal CA1 area. Taken together, these data underline the importance of PGC-1α in protecting against hippocampal neuronal damage under ischemic condition.

BCAS was used to induce chronic cerebral hypoperfusion in mice in our study. It was reported that this model could reasonably reproduce the clinical observations of VaD, and mimic some of the pathologies observed in the brains of VaD patients at autopsy 44. In addition, mitochondrial function in hippocampus was also proved to be impaired in the rat model of chronic cerebral ischemia 45. Our present findings is consistent with this report demonstrating that the levels of mitochondrial antioxidants including SOD2, Prx3, Trx2 and GPx1, and UCPs (UCP2, UCP4 and UCP5), in the hippocampus were significantly down-regulated in mice with chronic cerebral hypoperfusion. Correspondingly, the levels of PGC-1α mRNA and protein expressions also markedly decreased, which is in line with a postmortem study of MS patients, showing that PGC-1α is significantly decreased in cerebral cortex, and this decrease is correlated with neuronal loss 18. Importantly, a previous study indicated that the PGC-1α overexpression in a PD animal model protected against the MPTP-induced neuronal damage 17. Taken together, these observations strongly support that PGC-1α may play a critical role in the pathophysiology of VaD.

To better understand the role of PGC-1α in VaD, we generated the transgenic mice with the neuron-specific PGC-1α overexpression. We found that the PGC-1α overexpression improved the learning and memory abilities of mice after chronic cerebral hypoperfusion. Shibata et al. found that the damage of white matter appeared at day 14 after BCAS surgery 4, which also promoted cognitive impairment. We found that the PGC-1α overexpression partly relieved the demyelination. In addition, the knockdown of PGC-1α worsened the neurological outcomes, while the up-regulation of PGC-1α improved the cognitive impairment. All of these findings further confirmed the neuroprotective effects of PGC-1α on the BCAS model. Furthermore, LTP test indicated that PGC-1α improved the disrupted hippocampal synaptic plasticity, which is in line with a previous report that PGC-1α was involved in the formation and maintenance of hippocampal synapses 46. The biological processes enriched by GO analysis are also consistent with a previous finding demonstrating that PGC-1α protected against renal ischemia by regulating NAD biosynthesis and oxidative metabolism 47. In addition, several enriched signaling pathways were found to be related to cognitive function. Previous studies reported that the regulation for insulin signaling pathway could ameliorate cognitive impairment 48, 49. Our RNA-Seq data and Seahorse assay revealed that PGC-1α also improved the metabolic activity of neurons. The specific mechanism by which PGC-1α protects against the ischemia-induced cognitive impairment remains unknown. Recently, Wrann et al. demonstrated that the PGC-1α/FNDC5 pathway markedly induced hippocampal BDNF expression, leading to the enhanced cognitive function 50. In this present study, we observed that PGC-1α overexpression also induced hippocampal BDNF expression. And there was no statistically significant difference in the numbers of neurons, which is in line with the previous studies 4, 23. Actually, the neuronal loss in hippocampus obviously appeared at 8 months after BCAS surgery. In the BCAS model, we evaluated the neurological outcome at 4 weeks after this surgery. Thus PGC-1α may contribute to the neural recovery at the later stage. In addition, PGC-1α increased the expressions of ChAT, VAChT and ChT1. It was well documented that the cholinergic system played crucial roles in learning and memory functions 34. Taken together, our findings provide a molecular basis for the neuroprotective effects of PGC-1α on the cognitive impairment.

**Figure 9:**
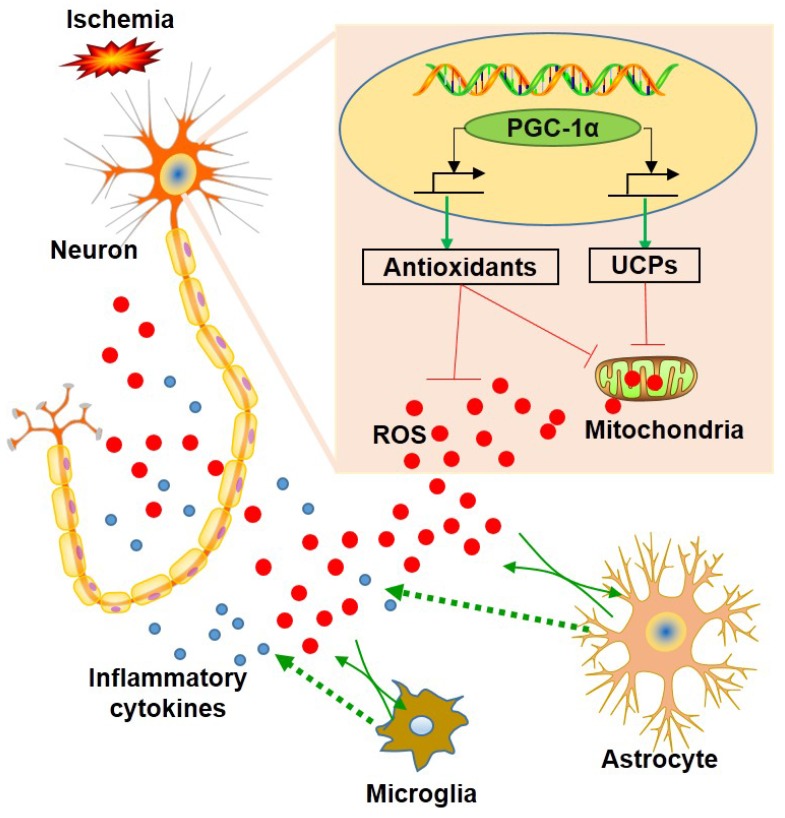
** Schematic representation of the role of neuronal PGC-1α in ischemia.** Ischemia induces reactive oxygen species (ROS) production from neurons and glia. In contrast, PGC-1α, a master regulator of mitochondrial function, is involved in mitochondrial biogenesis, and significantly up-regulates the expressions of mitochondrial antioxidants and uncoupling proteins (UCPs), thereby reducing the accumulation of ROS. Subsequently, this condition impedes glial activation, leading to decreased generation for ROS and inflammatory cytokines, and ultimately preventing from the neuronal dysfunction induced by chronic cerebral hypoperfusion.

A previous study revealed that the down-regulation of PGC-1α in neurons could lead to a significant reduction in mitochondrial antioxidants and UCPs 18. In this present study, we showed that the up-regulation of PGC-1α significantly increased the mRNA expressions of SOD2, Prx3, GPx1, UCP2, UCP4 and UCP5, which acts as an endogenous protective mechanism to suppress ROS production 51. In addition, DHE staining also confirmed that PGC-1α markedly reduced ROS level. Thus, we concluded that PGC-1α could enhance mitochondrial biogenesis by up-regulating mitochondrial antioxidants and uncoupling proteins, which in turn reduces the ROS-mediated neuronal damage. On the other hand, ROS is closely related to inflammatory responses, and neuroinflammation has been identified as an important mechanism that contributes to the pathophysiology of dementia 41, 52, 53. Previous studies also demonstrated that glial activation occurred in the hippocampus after chronic cerebral hypoperfusion 4, 6, 54, and the inhibition for microglia limited the neuronal damage in the hippocampus 55. In addition, the anti-inflammatory effects of PGC-1α have been well studied 56, 57. In our study, glial activation was down-regulated in the mice from the nPGC-1α+BCAS group, suggesting that PGC-1α exerts an anti-inflammatory effect. This study confirmed the important role of PGC-1α in regulating energy metabolism and the subsequent inflammatory reactions in hippocampus. Previous studies have demonstrated that increasing PGC-1α could effectively suppress microglia and astrocyte activations 58, 59. And ROS has been viewed as a powerful inducer to promote inflammatory responses by activating NLRP3 inflammasome or NF-κB pathway 60, 61. In this present study, PGC-1α significantly suppressed the production of ROS by promoting the expression of mitochondrial ROS-detoxifying enzymes and UCPs, and then the decreased ROS from neurons attenuated the activations of surrounding glial cells and suppressed the neuroinflammation in the BCAS model 62. However, the PGC-1α-modulated gene transcription and expression for the downstream process still need further experiments, including the measurement of target genes using chromatin immunoprecipitation assay. Collectively, our results supported the hypothesis that PGC-1α reduces ROS production, thereby reducing glial activation and improving cognitive dysfunction, as shown in Figure 9.

Clear evidences have indicated that hippocampal neurons were closely related to cognitive impairment 63, 64. PGC-1α, a transcriptional coactivator, was abundantly expressed in the tissues with high metabolic rates, especially in neurons and muscles 65. In addition, the specific role of PGC-1α was dependent on the transcription factors that are interacted with it 66. In this present study, we found that PGC-1α improved synaptic plasticity, the energy metabolism of hippocampal neurons, and promoted the expressions of BDNF and mitochondrial antioxidants, which all contributes to alleviate the cognitive impairment. Thus, it is convinced that PGC-1α plays crucial roles in the pathology of BCAS model. In addition, the neuroprotective effects of PGC-1α may not be limited to these points observed in this study. For instance, PGC-1α may participate in regulating autophagy, thereby affecting neuronal apoptosis. Thus, it is worth to continue to explore other action mechanisms of PGC-1α in the BCAS model in the future researches.

In summary, we showed that the PGC-1α overexpression in neurons protected against the cognitive impairment induced by chronic cerebral hypoperfusion. A possible molecular mechanism for this effect is that the neuronal PGC-1α powerfully reduces ROS production and suppresses the neuroinflammation under ischemic condition. Therefore, the neuronal PGC-1α may represent a promising therapeutic target for improving cognitive impairment after chronic cerebral hypoperfusion.

## Supplementary Material

Supplementary figures and table.

## Abbreviations

ACh: acetylcholine
AChE: acetylcholinesterase
AD: Alzheimer's disease
BCAS: bilateral common carotid artery stenosis
BDNF: brain-derived neurotrophic factor
BSA: bovine serum albumin
ChAT: choline acetyltransferase
ChT1: high-affinity choline transporter
DG: dentate gyrus
DHE: dihydroethidium
fEPSP: field excitatory postsynaptic potentials
GFAP: glial fibrillary acidic protein
GO: gene ontology
GPx1: glutathione peroxidase
Iba-1: ionized calcium-binding adaptor molecule-1
KEGG: kyoto encyclopedia of genes and genomes
LTP: long-term potentiation
MBP: myelin basic protein
MS: multiple sclerosis
MWM: Morris water maze
OCR: oxygen consumption rate
ODRL: odor discrimination reversal learning
OGD: oxygen and glucose deprivation
PD: Parkinson's disease
PGC-1α: peroxisome proliferator-activated receptor-γ co-activator 1α
PP: perforant pathway
Prx3: peroxiredoxin 3
ROS: reactive oxygen species
SOD2: superoxide dismutase 2
TBS: theta burst stimulation
Trx2: thioredoxin 2
UCPs: uncoupling proteins
NeuN: neuron-specific DNA-binding protein
VAChT: vesicle acetylcholine transporter
VaD: vascular dementia
