# Supplementary material for: Upregulation of neuronal PGC-1α ameliorates cognitive impairment induced by chronic cerebral hypoperfusion
Source: Theranostics. 2020 Feb 3;10(6):2832–48. doi: 10.7150/thno.37119 (PMC7052889; doi:10.7150/thno.37119)
Supplement: Supplementary file 1 — Supplementary figures and table. [file thnov10p2832s1.pdf]

Table S1. Primers used for qRT-PCR analysis.

Gene	Forward	Reverse
PGC-1α	ATGAATGCAGCGGTCTTAGC	GGTCATCGTTTGTGGTCAGA
SOD2	CAGACCTGCCTTACGACTATGG	CTCGGTGGCGTTGAGATTGTT
Prx3	GGTTGCTCGTCATGCAAGTG	CCACAGTATGTCTGTCAAACAGG
Trx2	TGGGCTTCCCTCACCTCTAAG	CCTGGACGTTAAAGGTCGTCA
GPx1	GTCTCTCTGAGGCACGATCCG	TTCCGCAGGAAGGTAAACAGC
UCP2	CAGGTCACTGTGCCCTTACCAT	CACTACGTTCCAGGATCCCAAG
UCP4	GAATGCCTATCGCCGAGGA	AGTAGGAACTTGCTCGTCCGG
UCP5	TCCCAACTGCTCAGCGTG	GGTGCTTCTTGGTAATATCATAAACG
GAPDH	TGTGATGGGTGTGAACCACGAGAA	CATGAGCCCTTCCACAATGCCAAA

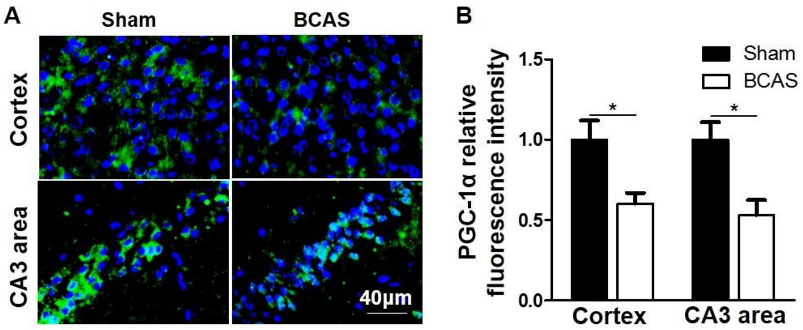

Figure S1. The expressions of PGC-1α are reduced in cortex and hippocampal CA3 areas of mice with chronic cerebral hypoperfusion.

(**A**) Representative images of immunofluorescent staining for the PGC-1α expressions in cortex and hippocampal CA3 areas of sham and BCAS groups. (**B**) Quantification of PGC-1α expression. *$p<0.05$ as determined by t Mann-Whitney *U* test. n = 6 in each group.

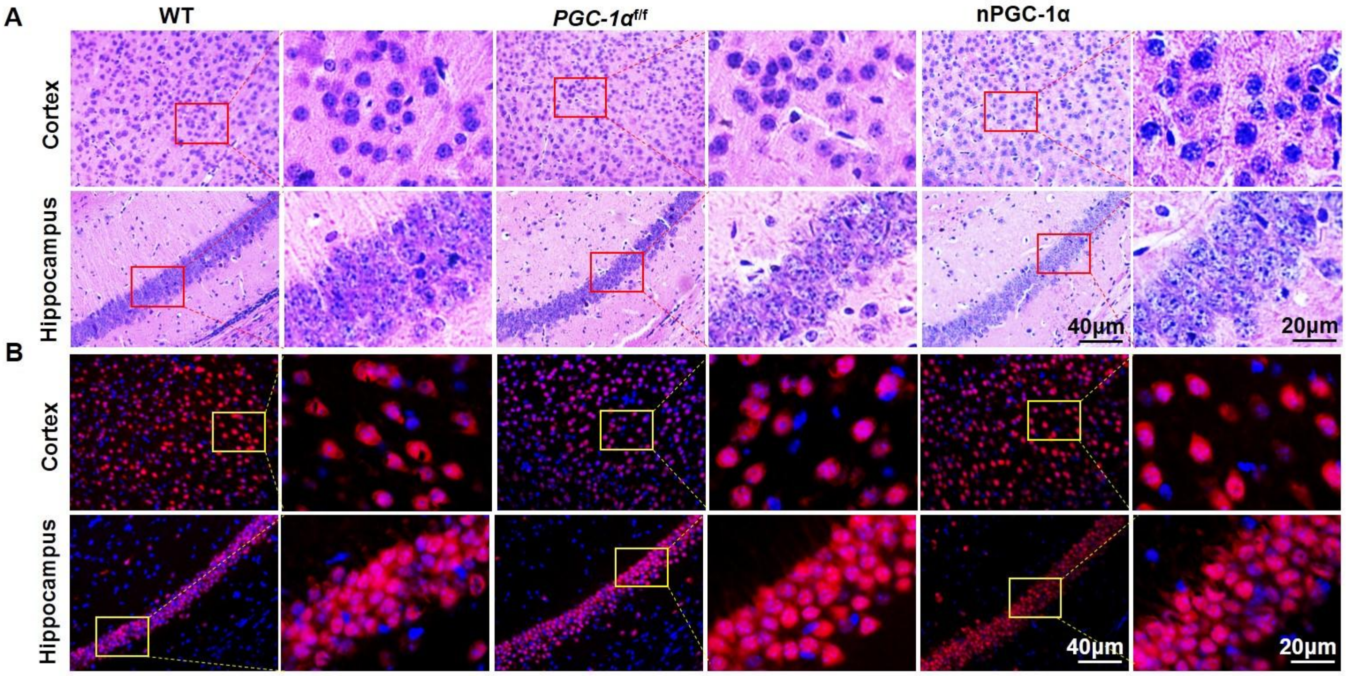

Figure S2. PGC-1α does not alter anatomical structure and the numbers of neurons.

(**A**) Representative images of H&E staining in cortex and hippocampal CA1 areas showed no abnormalities in WT, *PGC-1α*$^{f/f}$ or nPGC-1α groups. (**B**) Representative images of immunostaining for the NeuN-positive neurons in cortex and hippocampal CA1 areas. There were no obvious changes in neuron numbers among these 3 groups. n = 6 in each group.

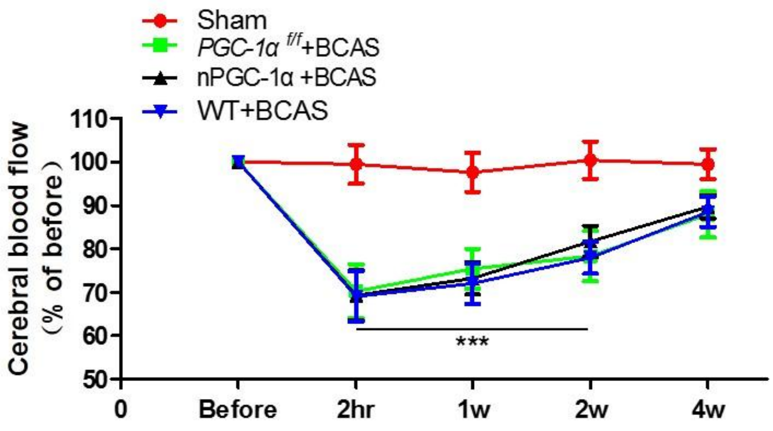

Figure S3. CBFs are down-regulated in 3 BCAS experimental groups in a similar manner.

CBF was recorded at different time points, including before and at 2 h, 1 week, 2 weeks and 4 weeks after BCAS surgery. CBF significantly decreased in these 3 experimental groups relative to the sham group. The decreased trend was similar among these 3 experimental groups. ***$p<0.001$ as determined by two-way ANOVA. n = 6 in each group.

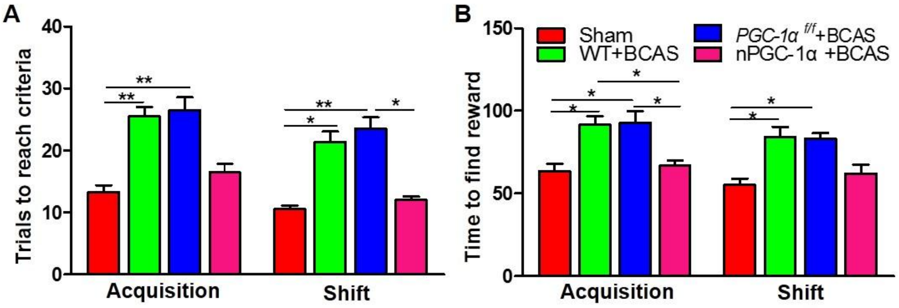

Figure S4. PGC-1α improves the executive function of mice with BCAS.

(**A**) Trials to reach criteria at the acquisition and shift stages of ODRL test for the sham, WT+BCAS, *PGC-1α*$^{f/f}$ +BCAS, and nPGC-1α+BCAS mice. (**B**) Time to get reward at the acquisition and shift stages of ODRL test. *p<0.05, **p<0.01 as determined by one-way ANOVA. n = 6 in each group.

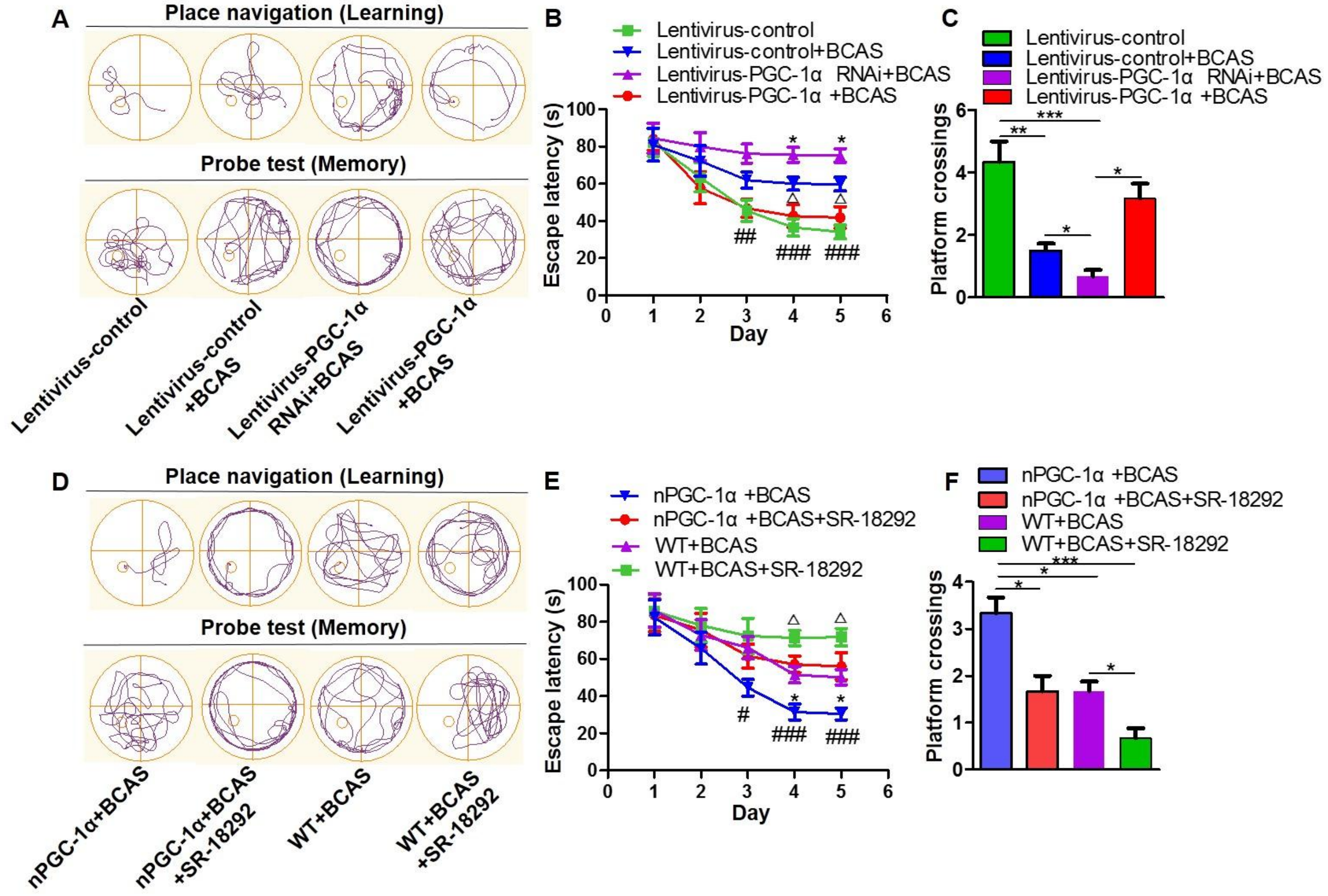

Figure S5. The regulation of PGC-1α expression alters the neurological outcomes in the BCAS mice model.

(**A**) The typical swimming paths for the mice from Lentivirus-control, Lentivirus-control+BCAS, Lentivirus-PGC-1α RNAi+BCAS, and Lentivirus-PGC-1α+BCAS groups in the MWM during learning (top) and memory probe tests (bottom). (**B**) Escape latencies significantly increased in the Lentivirus-control+BCAS and Lentivirus-PGC-1α RNAi+BCAS mice. *p<0.05 for the comparison between Lentivirus-PGC-1α RNAi+BCAS and Lentivirus-

control+BCAS groups; ##p<0.01, ###p<0.001 for the comparison between Lentivirus-PGC-1α RNAi+BCAS and Lentivirus-control groups; △p<0.05 for the comparison between Lentivirus-PGC-1α+BCAS and Lentivirus-PGC-1α RNAi+BCAS groups, as determined by two-way ANOVA. (**C**) The comparisons of mean numbers of platform crossings among 4 groups during the probe test. *p<0.05, **p<0.01, ***p<0.001 as determined by one-way ANOVA. (**D**) The typical swimming paths for the mice from nPGC-1α+BCAS, nPGC-1α+BCAS+SR-18292, WT+BCAS, and WT+BCAS+SR-18292 groups in the MWM during learning (top) and memory probe tests (bottom). (**E**) Escape latencies significantly increased in the WT+BCAS+SR-18292 mice. *p<0.05 for the comparison between nPGC-1α+BCAS+SR-18292 and nPGC-1α+BCAS groups; #p<0.05, ###p<0.001 for the comparison between WT+BCAS+SR-18292 and nPGC-1α+BCAS groups; △p<0.05 for the comparison between WT+BCAS+SR-18292 and WT+BCAS groups, as determined by two-way ANOVA. (**F**) The comparison of mean numbers of platform crossings during the probe test. *p<0.05, ***p<0.001 as determined by one-way ANOVA. n = 6 in each group.

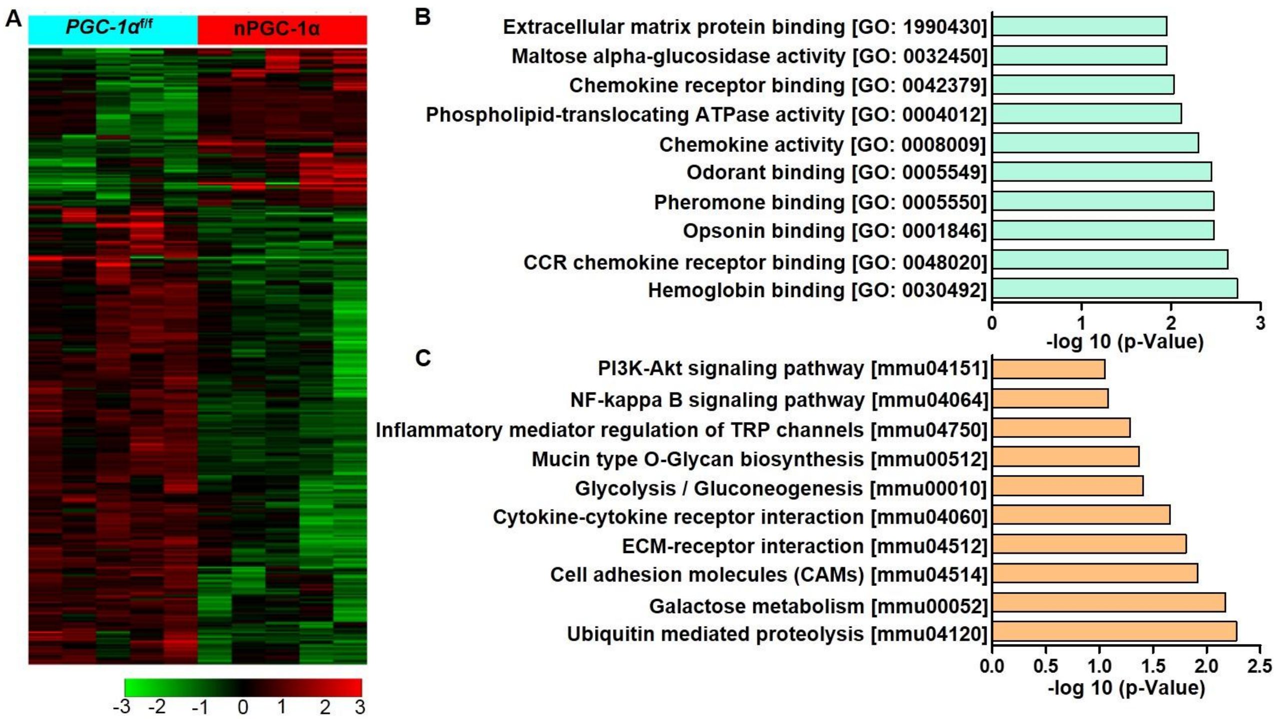

Figure S6. PGC-1α alters the gene expression profiles in hippocampus under basal conditions.

(**A**) Cluster analysis for the differentially expressed mRNAs in hippocampus between *PGC-1α*$^{f/f}$ and nPGC-1α groups were performed using microarray. One hundred up-regulated and 290 down-regulated genes were identified. (**B**) Go analysis showed the top 10 enriched molecular functions. (**C**) KEGG analysis showed the top 10 enriched pathways. n

= 5 in each group.

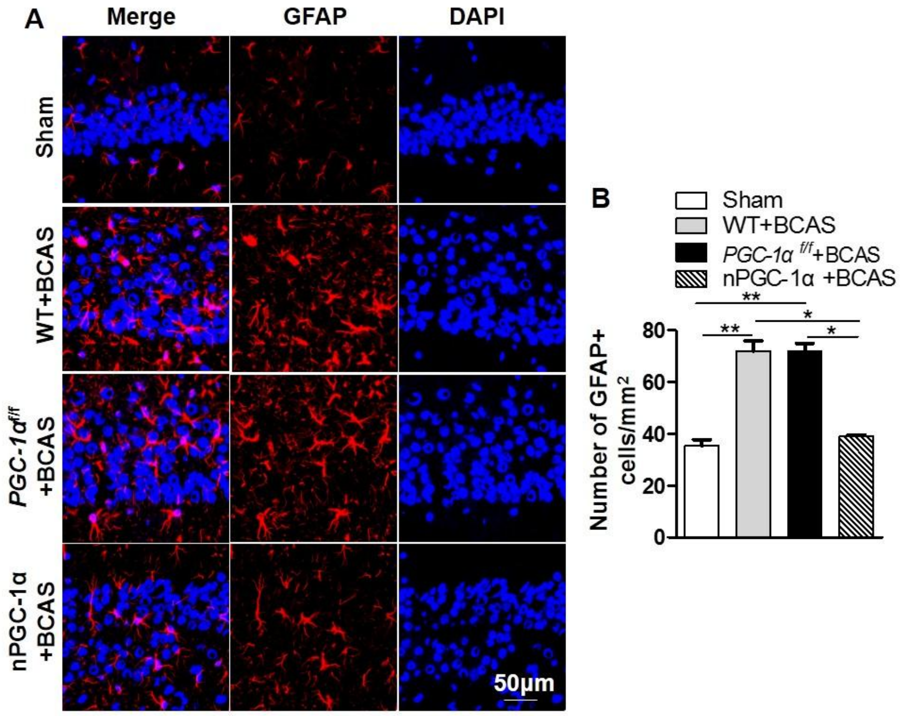

Figure S7. PGC-1α reduces the activation of astrocytes.

(**A**) Representative images of the GFAP-positive astrocytes in hippocampal CA1 areas in WT+BCAS, *PGC-1α*$^{f/f}$ +BCAS, nPGC-1α+BCAS and sham mice. (**B**) The numbers of the GFAP-positive astrocytes. *p<0.05, **p<0.01 as determined by one-way ANOVA. n = 6 in each group.

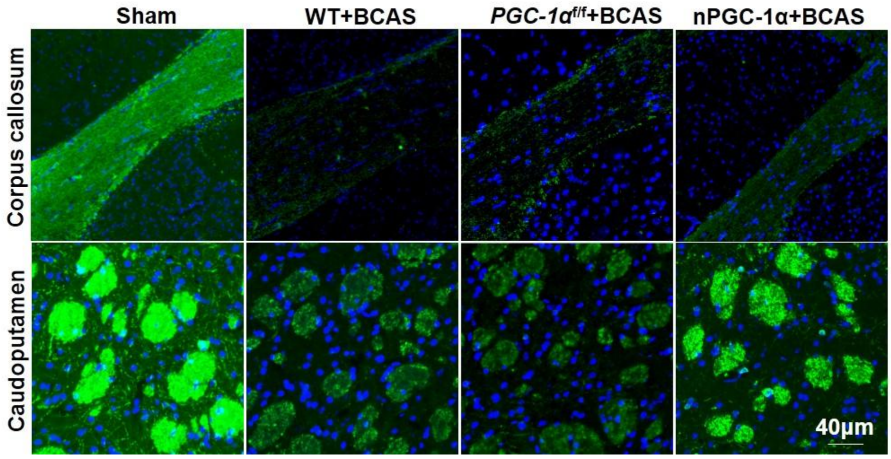

Figure S8. PGC-1α attenuates the damage of white matter in BCAS mice.

(**A**) Representative images of immunofluorescent staining for MBP in corpus callosum from the sham, WT+BCAS, *PGC-1α*$^{f/f}$ +BCAS, and nPGC-1α+BCAS mice. (**B**) Representative images of immunofluorescent staining for MBP in caudoputamen.
